# High Costs of Dialysis Transportation in the United States: Exploring Approaches to a More Cost-effective Delivery System

**DOI:** 10.36469/9861

**Published:** 2013-08-28

**Authors:** J. Mark Stephens, Samuel Brotherton, Stephan C. Dunning, Larry C. Emerson, David T. Gilbertson, Matthew Gitlin, Ann C. McClellan, William M. McClellan, Sanatan Shreay

**Affiliations:** 1 Prima Health Analytics, Boston, MA; 2 Chronic Disease Research Group, Minneapolis, MN; 3 Dialysis Center of Lincoln, Lincoln, NE; 4 Amgen Inc., Thousand Oaks, CA; 5 Emory University, Atlanta, GA

**Keywords:** end-stage renal disease, cost-effective, costs, transportation, dialysis

## Abstract

**Background:** The costs of transporting end-stage renal disease (ESRD) patients to dialysis centers are high and growing rapidly. Research has suggested that substantial cost savings could be achieved if medically appropriate transport was made available and covered by Medicare.

**Objectives:** To estimate US dialysis transportation costs from a purchaser’s perspective, and to estimate cost savings that could be achieved if less expensive means of transport were utilized.

**Methods:** Costs were estimated using an actuarial model. Travel distance estimates were calculated using GIS software from patient ZIP codes and dialysis facility addresses. Cost and utilization estimates were derived from fee schedules, government reports, transportation websites and peer-reviewed literature.

**Results:** The estimated annual cost of dialysis transportation in the United States is $3.0 billion, half of which is for ambulances. Most other costs are due to transport via ambulettes, wheelchair vans and taxis. Approximately 5% of costs incurred are for private vehicle or public transportation use. If ambulance use dropped to 1% of trips from the current 5%, costs could be reduced by one-third.

**Conclusions:** Decision-makers should consider policies to reduce ambulance use, while providing appropriate levels of care.

## BACKGROUND

Among the chronically ill, travel for medical care can be burdensome from both a time and cost perspective, and may be an important factor in treatment decisions and patient outcomes.[1–3] Travel costs may be especially high for people with end-stage renal disease (ESRD) who receive hemodialysis, as these patients typically have to travel for outpatient treatment three times per week, every week of the year, generally for the remainder of their lives. The high cost of transport for hemodialysis patients is not only due to the frequency of travel, but also driven by the physical assistance needs of this population. Dialysis patients have an average of 3.2 comorbidities,[4] 51% are over age 65,[5] over one-third use a wheelchair or a walker and half require some type of mobility device.[6]

Due to the high prevalence of diabetes in this population, many are amputees. At least half are transportation dependent, meaning they cannot drive themselves and are unable to take public transit.[6]

The costs of transporting this very ill, elderly and largely non-ambulatory population for medical care are high and growing rapidly. In 2009, Medicare spent $930 million on ambulance transports alone for ESRD patients;[7] a 60% increase over 2005 expenditures. Patients or secondary insurers paid an additional 25% or another $230 million, for a total cost of nearly $1.2 billion in 2009. Medicare's recent cost containment efforts in the ESRD program have largely focused on outpatient dialysis and injectable drugs. However, total expenditures for these services increased by only about 4% per year between 2005 and 2010.[5] During this same period, the cost of ambulance transports for ESRD patients rose at an average annual rate of 15%.[7]

Medicare does not pay for any transportation services except ambulances, which are only covered when deemed medically necessary. Therefore, while cost containment efforts have focused primarily on services with low growth trends, the issue of the use of ambulances for transport to routine dialysis has been identified, but not adequately addressed, for at least the past two decades. As early as 1994, the Office of the Inspector General (OIG) was charged with investigating the medical necessity of, and payment practices related to ambulance transportation of dialysis patients. The OIG found that less than 2% of ESRD beneficiaries accounted for 75% of total ESRD ambulance payments.[8] The high expense for so few people was related to the routine use of ambulances for trips to dialysis facilities. OIG also found that 70% of dialysis-related ambulance claims examined did not meet Medicare coverage guidelines for medical necessity because beneficiaries did not have conditions that contraindicated use of another type of transport.[9] A subsequent General Accounting Office (GAO) report showed that more than 50% of rural Medicare ambulance trips were of a non-emergency nature.[10]

Other research has suggested that substantial cost savings could be achieved if medically appropriate transport was made available and covered by Medicare. Burkhardt[11] demonstrated that Medicare's transportation policy was not cost-effective, with Medicare often paying for ambulance trips when less expensive, alternative transportation could have been used. Further, Medicare could have saved 96% of what it paid for ambulance transports that were subsequently determined to be non-emergencies. The paper noted that cost was not the only burden that would be relieved if Medicare paid for appropriate types of non-emergency medical transportation (NEMT), as this would greatly simplify the logistics for patients, state programs and health providers. Finally, a 2012 report by the Medicare Payment Advisory Committee (MedPAC) showed that reducing ambulance use for dialysis transport in high-use states to the national median would save $400 million per year.[12] However, these findings and the previous reports from the OIG and GAO did not lead to new legislation that would address the fundamental problem of the lack of Medicare coverage for less expensive alternative transportation.

Given the high costs and rapid growth in the costs of dialysis transportation and the well-documented problems in managing these costs in the Medicare program, the paucity of recent research examining the extent and nature of such costs and their policy implications needs to be addressed. The total annual costs of dialysis transportation in the United States are difficult to estimate. To our knowledge, no comprehensive studies or estimates have been published to date. While estimates are available reporting Medicare costs for ambulance transportation for dialysis patients, as are annual NEMT costs (of which some substantial portion is for dialysis patient transport), transportation costs for other public assistance programs available to dialysis patients and those borne by private insurers and patients and their families are more difficult to estimate, and may be higher than the payments made by Medicare or Medicaid.

Therefore, the objectives of this study were (1) to estimate US dialysis transportation costs from a purchaser's perspective, in total, by transport and payer type and by state and region, and (2) to estimate potential cost savings that could be achieved if less expensive means of transport were utilized.

## METHODS

### Data Sources and Approach

This was a retrospective modeling study of dialysis patient travel estimates and the costs of travel, using publicly available data on U.S. dialysis patients and facilities in 2012. Using an actuarial model, the total costs of transportation were estimated for about 360 000 patients for travel to routine in-center dialysis. Per trip travel distance estimates were calculated from patient ZIP codes and dialysis facility addresses. Costs were estimated from a purchaser's perspective, where costs were defined as the total direct financial costs of all types of dialysis transportation incurred by the purchaser(s) of the service. This was either payments made by insurers, patients or other third parties to the transport provider for the actual services used, or, where no transport provider was paid for the transportation service, (i.e., patients drove themselves by private auto), a direct cost was calculated based on standard mileage rates.

Transit service to a dialysis facility is provided by a variety of modes, defined by the type of vehicle used, operating characteristics of the service provided, and the travel needs of the riding public for which they are designed. The transportation categories used in the model are defined in Table 1. Utilization by transport type used to travel to dialysis and costs of each transport type were estimated from fee schedules, government reports, transportation websites and peer-reviewed literature. The primary data sources and assumptions used in the model are summarized in Table 2.

### Model Design

The model used was an actuarial-type deterministic model, where all inputs were pre-defined, with predetermined application of formulae to the inputs to produce output. Costs were estimated for all in-center dialysis patients in the 50 US states, the District of Columbia and Puerto Rico. The base year for estimates was 2012. Costs were projected forward to 2014 using transportation cost inflation trends[13] and dialysis patient growth trends.[7]

Costs were estimated using the following input variables:

- Number of patients;
- Average number of treatments per patient per year (PPPY);
- Distance traveled per treatment; and
- Cost of travel per mile/treatment.

Input variables were detailed by the following dimensions:

- Insurance category (e.g., Medicare, Medicaid, commercial, no insurance);
- Transport type (e.g., ambulance, wheelchair van, private auto);
- Patient location (e.g., state, urban versus rural); and
- Calendar year.

**Table 1.** Definitions for Transport Service Categories Used in the Model

| Transport Type | Defenition |
|---|---|
| **Non-emergency Medical Transportation** | A general term for any type of transportation provided for transport of patients to medical appointments on a non-emergency basis. Transport services utilized for NEMT range from ambulances to private autos. |
| **Paratransit** | General term for assisted transport for persons with disabilities. Paratransit vehicles take passengers directly from their origins to their destinations, usually by van. By law, accessible paratransit service must be provided in all areas served by regular route transit service to persons with disabilities or otheriwse unable to use fixed-route service. Paratransit service is often open to larger segments of the public or all riders. Some services are operated during late night and weekend hours in place of fixed-route services.[31] |
| **Ambulance** | Vehicle equipped with medications and devices intended to stabilize patients while speeding them to emergency care. |
| **Ambulette (Stretcher Van)** | Van specifically squipped for the purpose of providing NEMT. Ambulettes are built with layouts that are conducive to handling a wide variety of wheelchair sizes and are often equipped with oxygen holding brackets, stretcher munts and wheelchair storage systems. |
| **Wheelchair Van (Paratransit Van, Chair Car)** | These terms refer to vans that can accomodate wheelchairs, but typically are designed to handle a mix of wheelchair users and other passengers. They are distinguished from ambulettes in that a simple wheelchair van is not specifically equipped for medical transportation. They are often used by county services for the elderly and disabled, and by public transit systems that need to provide services to poeple with physical disabilities. |
| **Taxi** | Commercial auto or van services that are typically hired on a per trip basis for transport to and from a single starting and ending point on as-needed basis. Taxis are often used to transport dialysis patients from hoe to the dialysis center and back with pick-up and drop-off timed to the dialysis session appointment time. |
| **Fixed-route Public Transportation** | This category encompasses most public transit systems of buses, subways, trolleys, ferries, and passenger rail systems that run on fixed routes and fixed schedules. It also includes paratransit services provided by public transit systems. |
| **Self-transport** | This refers to patients who drive themselves to or from dialysis. |
| **Other Divers** | This category covers other types of transport for ambulatory patients not needing wheelchair or medical services. Includes private autos of friends and family, van pools, etc. |

Model outputs were:

- Number of patients,
- Number of treatments,
- Distance traveled,
- Costs of travel, and
- Sums and averages of the above: US total, PPPY, per treatment.

**Table 2.** Summary of Data Sources and Assumptions Used for Estimating Transport Costs

| Category | Source(s) | Assumptions |
|---|---|---|
| **Population** | | |
| 2012 dialysis patient counts by ZIP code and modality | National Patient Prevalence Report, from the ESRD Networks[32] | Point in time patient count. Assumed to be the average from all months in 2012 |
| Patient proportions by insurance category | 2011 USRDS Annual Data Report,[7] checked agains statistics aggregated from 2009 Medicare Renal Cost Reports[33] | Detailed non-Medicare estimates were interpolated from 2011 Dialysis Facility Reports[4] |
| **Utilization** | | |
| Average number of annual treatments per patient | 2009-2010 Medicare Cost Reports[17,18] | Used average of 209-2010 for hemodialysis patients only |
| Average travel distance to treatment | Patient by ZIP code data from the ESRD Networks National Pervalence Report[32] and facility location data from the Dialysis Facility Compare dataset[34] | See methods description under Patient Travel Estimates |
| Mode of transport: Number/percent of annual trips to dialysis via ambulance transport | 2009 Medicare ambulance costs PPPY from the 2011 USRDS Annual Data Report,[7] 2009 Medicare Ambulance Fee Schedules,[35] and average miles to treatment estimates from this study | An average cost per trip was calculated for each state, urban, rural and super-rural. Cost PPPY was divided by cost per trip to estimate total trips PPPY |
| Estimated proportions of travel via other modes of transport | 2002-2003 National Survey of Medicaid Non-emergency Transportation,[36] and other published reports and modeling | Assumptions varied by insurance category, state and urban versus rural |
| **Costs** | | |
| Ambulance costs per trip | CY 2012 Medicare Ambulance Fee Schedules,[37] in combination with the travel mile estimates from this study | Assumed Basic Life Support (BLS) level of transport |
| Capitation costs for dialysis transportation for Medicare Advantage patients | Estimated from the 2012 United States Per Capita Costs for dialysis patients[38] | Assumed capitation costs were equivalent to Fee-for-Service as percentage of total |
| Per mile costs for private transportation | Based on 2012 IRS mileage rates[39] | Medical rate applied to self-transported miles. Full business rate applied to other mileage-based cost estimates |
| Costs of other modes of transport | Estimated from Medicaid program survey, government reports, rate schedules for taxis, buses, and other public transport services and published literature on transport costs and models | Used national averages, split by urban versus rural |
| Transportation cost inflation | Based on the Urban Consumer Price Index (CPI-U for Transportation, published by the Bureau of Labor Statistics[13] | Used average of last 3 years to project to 2014 |
| Cost burden for each payer category | Estimated form review of plan benefit descriptions for Medicare, Medicaid and select commercial insurance plans | Assumed patients without insurance pay charges |

ESRD=end-stage renal disease; IRS=internal revenue service; PPPY=per patient per year; USRDS=United States renal data system

Output variables were developed in detail by:

- Insurance category,
- Transport type,
- Patient location,
- Calendar year, and
- Payer (e.g., Medicare, Medicaid, private insurance, patient/other).

The full matrix of detailed cost estimates across all model dimensions included thousands of data points. Each data point was estimated using actuarial techniques through a combination of detailed estimates and higher level adjustment factors. For example, an average cost per trip for ambulettes was modified by an insurance-type factor to differentiate what Medicaid would pay versus what a patient would pay out-of-pocket. Detailed utilization estimates (i.e., treatments, transport miles) by transport type and insurance category were also calculated by applying utilization proportions to the detailed estimates of patients, miles, and visits that were derived from the distance calculations. The output from distance calculations was miles per treatment and patient counts by ZIP code.

Each detailed estimate was then broken down by insurance category and transport type based on higher level estimates for the general population. A further delineation of each cost estimate was made by applying payment allocation factors to each insurance category-by-transport type, reflecting the insurance benefits for each transport type in each insurance category. For example, Medicare pays 80% of the allowable costs of ambulance services. The remainder is paid by third parties such as Medicaid (for dual eligible beneficiaries), private insurance or the patients themselves.

**Patient Travel Estimates (distance calculations)**

To estimate patient travel distances for dialysis, two data points were estimated: (1) patient residence and (2) dialysis treatment destination. These data points were mapped to latitude and longitude coordinates using Microsoft MapPoint 2011 and GPS Visualizer software. Driving distances and times were calculated using custom software developed in MapPoint and Wolfram Mathematica 8. Patient address data were limited to ZIP code of residence. Patients were assigned to residential locations within their ZIP code, weighted by population density. For example, an urban center within a ZIP code received a higher allocation of patients than a rural area of the same size within the same ZIP code.

Data on each patient's actual facility were not obtained. It was assumed that the facility location closest to the patient residence was the one utilized. This assumption is commonly used in studies of dialysis access.[14–16] However, initial modeling of patient assignments to their nearest facility revealed that such an assumption produced unrealistic patient counts at many facilities. Many facilities would be heavily over-or under-allocated, relative to the actual number of patients served by these facilities. To address this problem, the model's initial patient assignment results were compared with reported patient census counts from Medicare Cost Reports[17,18] and Dialysis Facility Reports,[4] and the model-assigned patient counts were "corrected" by randomly selecting patients from over-allocated facilities and re-assigning them to other nearby facilities so that the number of patients assigned to each facility in the model achieved a 90% correlation with the actual patient counts reported by facilities.

Comparisons with point estimates found in recent literature on U.S. patient travel for dialysis services[19–21] were made to validate the estimates of patient travel distance and time to dialysis facilities made for this study.

## Statistical Analysis

### *Study Outcomes*

Transportation costs, trips and miles were estimated nationally, by state, census region and ESRD network for urban, rural and super-rural subgroups, by transport type and payer. Rates were calculated for average costs per patient, per round-trip and per mile. Costs were cumulated over 3 years —2012 through 2014.

### *Reliability and Validity*

Three sets of comparisons were made between model estimates and other cost estimates from the literature: (1) total national costs of dialysis transportation, (2) Medicare payments for ambulances, and (3) NEMT costs per trip. In general, the estimates from this study were conservative by comparison to the other estimates available from the literature. Model estimates were 7% to 12% lower than the comparison figures. For example, the model estimate of 2012 Medicare ambulance payments per patient for routine dialysis transportation was approximately 8% lower than those calculated from the 2009 actual Medicare ambulance payments (trended for inflation).

### *Cost Saving Scenarios*

In addition to the baseline estimates, cost estimates and potential cost savings were calculated for three scenarios in which utilization of higher cost means of transport was shifted to less expensive transport types. The three scenarios tested were:

- **Scenario 1:** Reduce ambulance utilization to 1% of trips. In the baseline model, seven states had ambulance use below 1% of total trips. This scenario calculated the savings that would result if all states had ambulance utilization of 1% of trips, and excess ambulance trips were proportionally allocated to all other modes except self-transport.
- **Scenario 2:** Change the mix of non-ambulance commercial NEMT transports (i.e., ambulettes, wheelchair vans, taxis and public transit) to a lower cost mix based on local "best practices". In the baseline estimates, the national average mix of transports within these four categories was 7%, 54%, 29%, and 10%, respectively. The mix used in this scenario was 5%, 25%, 60%, and 10%, respectively. It was assumed that public transit use could not be higher than the national average, due to limited availability in rural areas and access problems for most dialysis patients. Reducing ambulette and wheelchair van use to 30% of this total from the national average of 61% may be aggressive when compared to current practices. It was assumed that some ambulette trips could be shifted to wheelchair vans, and most wheelchair van trips could be shifted to taxis.
- **Scenario 3:** Combine the utilization shifts from Scenarios 1 and 2, first shifting excess ambulance use proportionally to all other modes except self-transport, and then shifting the mix within commercial NEMT to the proportions in Scenario 2.

### *Sensitivity Analysis*

A series of one-way sensitivity analyses were conducted for the 3-year (2012-2014) projected costs of dialysis transportation for in-center dialysis patients, to determine the key cost drivers estimated in the model. Eight variables expected to show sensitivity in the results to changes in their values were identified in the model. The effects of changes from the baseline assumption for each single variable in the analysis were isolated in the model by holding all other factors constant (at baseline estimates) and then modifying the test variable through a range of reasonable alternate assumptions. Model results for total (cumulative) costs under these alternate assumptions were compared to the baseline result. Plausible ranges of values were developed from the literature and/or from sub-analyses to bound the one-way sensitivity analyses. Sources and rationales for the assumptions used are given in Table 3.

**Table 3.** Sensitivity Analysis: Plausible Ranges of Values, Sources and Rationales

| Variable | Lower Boundary | Baseline | Upper Boundary |
|---|---|---|---|
| **Utilization by Transport Type[a]** | West | US Average | Northeast |
| **NEMT Cost per Trip[b]** | \$24.22 | \$30.28 | \$36.34 |
| **Insurer Discounts[c]** | Higher Commercial/ Medicaid Discounts | Best Estimate Current Discounts | Non-Medicare at Charges |
| **Travel Miles (median miles/trip)[d]** | 4.2 | 4.2 | 6.0 |
| **Annual Treatments/Patient[e]** | 137 | 142 | 146 |
| **Milleage Allowance (\$/mile)[f]** | \$0.230 | \$0.388 | \$0.555 |
| **Patient Insurance Distribution[g]** | Good Economy | Best Estimate (2009) | Poor Economy |
| **Annual Transportation Inflation Rate[h]** | 5.6% | 6.8% | 8.3% |

NEMT=non-emergency medical transportation

[a] Range based on U.S. regions with the least intensive (West) and most intensive (Northeast) mix of transportation services utilized in the model.

[b] Few NEMT cost estimates per trip were found in the literature. A range of ±20% around the baseline estimate was used.

[c] Low end: Commercial NEMT discounts same as Medicaid, ambulance fees at Medicare fee schedule for commercial and Medicaid. High end: no discounts, Medicaid or Commercial.

[d] Low end estimate: Baseline model, which was the lowest estimate found. High end estimate: MedPAC 2011 Report to the Congress, which examined travel for new Fee for Service beneficiaries in 2008.[19]

[e] Low estimate from average of 2009 Renal Cost Reports[17] for in-center hemodialysis + peritoneal dialysis. High estimate from USRDS 2011 Annual Data Report[7]
Table J.8 for 2009 (including hospitals). Base estimate was the average of 2009-2010
hemodialysis-only from all cost reports (includes hospital non-maintenance sessions).

[f] Low estimate: All miles at Internal Revenue Service (IRS) 2012 Medical Rate (variable costs only). High estimate: All miles at IRS 2012 full mileage rate (all costs included).[39]

[g] Based on economic scenarios of poor economy = more Medicaid/more uninsured/less commercially-insured, and good economy = less Medicaid/less uninsured, more commercially-insured. Assume Medicare eligible patients stay about the same.

[h] Used the high and low annual percentage inflation of the last 5 years of the CPI-U Transportation Index[13] and the high and low annual percentage growth in dialysis patient year-end point prevalence of the most recent 5 years available (2004 to 2009).[7] The baseline assumption was 6.8% annual growth, which was the average of the past 3 years.

## RESULTS

The study cohort used in the model included 360 579 in-center dialysis patients assigned to 5405 dialysis centers (Table 4).

**Table 4.** Study Cohort of Patients and Facilities

| Dialysis Patients | n | Dialysis Facilities | n |
|---|---|---|---|
| Total US dialysis patients[a] | 401 109 | Total US ESRD providers[b] | 5681 |
| Home dialysis modalities | -40 103 | No in-center dialysis services | -215 |
| In-center dialysis patients | 361 006 | In-center dialysis providers | 5466 |
| Missing data[c] | -427 | No patients assigned[d] | -61 |
| Patients in results | 360 579 | Facilities in results | 5405 |

ESRD=end stage renal disease
[a]National Patient Prevalence Report32
[b]Centers for Medicare & Medicaid Services (CMS) Dialysis Facility Compare File34
[c]These patients could not be assigned to a dialysis facility, since the model allocated them to geographically inaccessible areas (e.g. wilderness areas and bodies of water).
[d]61 facilities had no patients assigned as the closest facility

The estimated annual cost of transportation to dialysis in the United States for 2012 was $3.0 billion, approximately half of which was for ambulance transport. Most of the remaining costs were for use of commercial transport via ambulettes, wheelchair vans and taxi services. Only about 5% of costs were related to use of private vehicles or public transportation. Costs were expected to grow to $3.4 billion by 2014 (Table 5).

**Table 5.** Estimated National Costs of Dialysis Transportation from 2012 to 2014, by Transport Type ($ Millions)

| Transport Type | 2012 | 2013 | 2014 |
|---|---|---|---|
| Ambulance | $1448.00 | $1546.60 | $1651.90 |
| Ambulette | $345.40 | $368.90 | $394.10 |
| Wheelchair van | $823.30 | $879.40 | $939.30 |
| Taxi | $225.60 | $241.00 | $257.40 |
| Fixed-route public transport | $9.10 | $9.70 | $10.30 |
| Self-driven | $42.60 | $45.50 | $48.60 |
| Other drivers | $111.00 | $118.60 | $126.70 |
| Total | $3005.00 | $3209.70 | $3428.30 |

We estimated that in-center dialysis patients would travel almost 744 million miles to dialysis in 2012, an average of 14.5 miles per round-trip. Collectively, they would make an estimated 51.2 million round-trips for dialysis in 1 year, an average of 142 round-trips per patient (Table 6). Ambulance services account for 48% of all costs, but only 5% of transports. Patients who drive themselves account for 25% of all trips, but only 1% of costs.

**Table 6.** 2012 National Utilization Statistics - Transportation to In-Center Dialysis

| Transport Type | Round Trips | Miles Traveled | % Costs | % Trips | % Miles | Round-trips/ Patient | Miles/ Round-trip |
|---|---|---|---|---|---|---|---|
| Ambulance | 2 739 361 | 39 267 051 | 48% | 5% | 5% | 8 | 14.3 |
| Ambulette | 1 681 214 | 27 155 597 | 11% | 3% | 4% | 5 | 16.2 |
| Wheelchairvan | 12 347 608 | 164 621 013 | 27% | 24% | 22% | 34 | 13.3 |
| Taxi | 6 773 622 | 85 619 569 | 8% | 13% | 12% | 19 | 12.6 |
| Fixed Route Public Transport | 2 368 816 | 30 992 212 | 0% | 5% | 4% | 7 | 13.1 |
| Self-driven | 12 758 287 | 185 242 492 | 1% | 25% | 25% | 35 | 14.5 |
| Otherdrivers | 12 533 271 | 210 926 863 | 4% | 25% | 28% | 35 | 16.8 |
| Total | 51 202 180 | 743 824 797 | 100% | 100% | 100% | 142 | 14.5 |

Costs PPPY were estimated at over $8300. About one-third of these costs would not be covered by patients' primary or secondary insurance (Medicare, Medicaid, or Employer Group Health Plans) and would therefore be the responsibility of the patient or other third parties (Table 7). The costs to Medicare for ambulances alone was expected to exceed $1.0 billion in 2012, not including emergency ambulance services for these patients.

**Table 7.** 2012 Costs ($ Millions) and Percent of Costs by Transport Type, by Payer

| Transport Type | Medicare | | Medicaid | | Private Insurance | | Patient/Other | | Total All Payers | |
|---|---|---|---|---|---|---|---|---|---|---|
| Ambulance | $1060.90 | 73% | $168.80 | 12% | $38.20 | 3% | $180.10 | 12% | $1448.00 | 100% |
| Ambulette | $0.00 | 0% | $153.50 | 44% | $26.20 | 8% | $165.70 | 48% | $345.40 | 100% |
| Wheelchair van | $0.00 | 0% | $396.60 | 48% | $87.00 | 11% | $339.70 | 41% | $823.30 | 100% |
| Taxi | $0.00 | 0% | $73.60 | 33% | $0.00 | 0% | $152.00 | 67% | $225.60 | 100% |
| Fixed route public transport | $0.00 | 0% | $3.20 | 35% | $0.00 | 0% | $5.90 | 65% | $9.10 | 100% |
| Self-driven | $0.00 | 0% | $14.20 | 33% | $0.00 | 0% | $28.40 | 67% | $42.60 | 100% |
| Other drivers | $0.00 | 0% | $13.40 | 12% | $0.00 | 0% | $97.70 | 88% | $111.00 | 100% |
| Total | $1060.90 | 35% | $823.40 | 27% | $151.40 | 5% | $969.40 | 32% | $3005.10 | 100% |

Per patient dialysis transportation costs vary tremendously from state to state, ranging from $3951 PPPY in Utah to $30 516 PPPY in Puerto Rico. As at the national level, costs by state were driven by ambulance utilization. The percentage of trips via ambulance in Utah was less than 1%, while it was 47% in Puerto Rico (Figure 1).

**Figure 1.** Dialysis Transportation Cost PPPY and Percent of Trips by Ambulance, by State

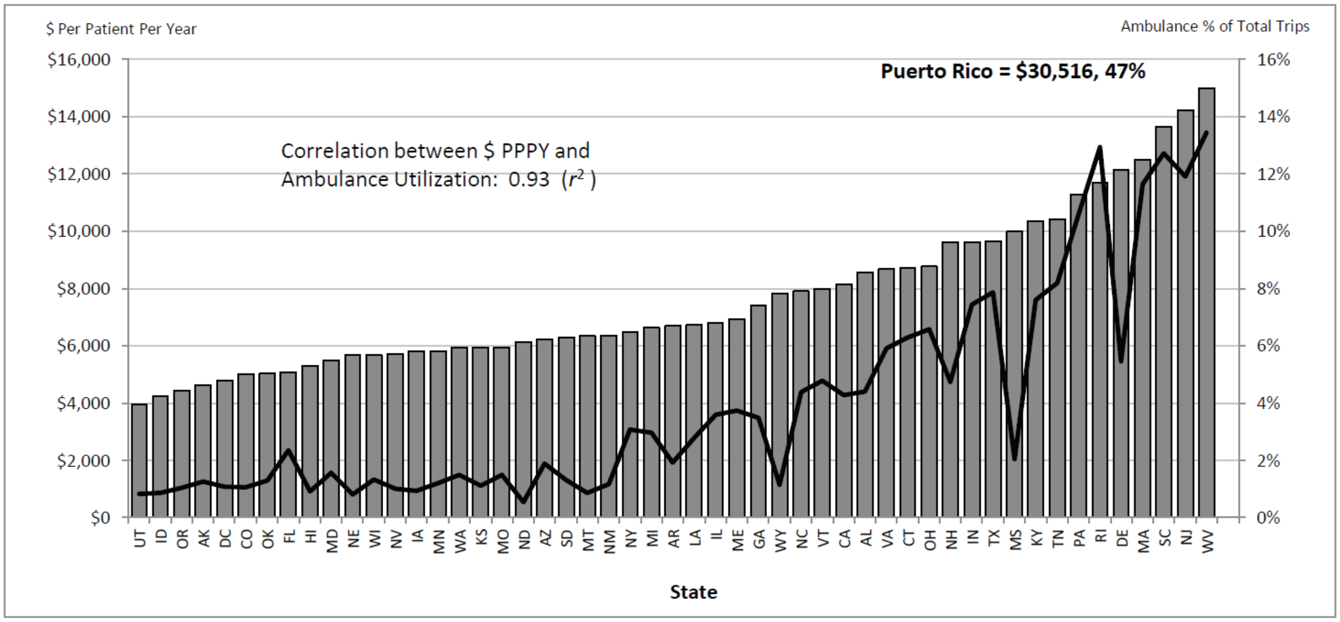

The correlation was calculated from state-level observations, using the percent of total trips that were via ambulance correlated with total costs PPPY.

There was also high regional variation, with PPPY costs of $7,119 in the Midwest versus $9780 in the Northeast (Figure 2). The percentage of costs attributed to ambulances was 39% in the Midwest and 56% in the Northeast.

**Figure 2.** Dialysis Transportation Costs PPPY and Percent of Costs Attributable to Ambulance Trips, by Census Region

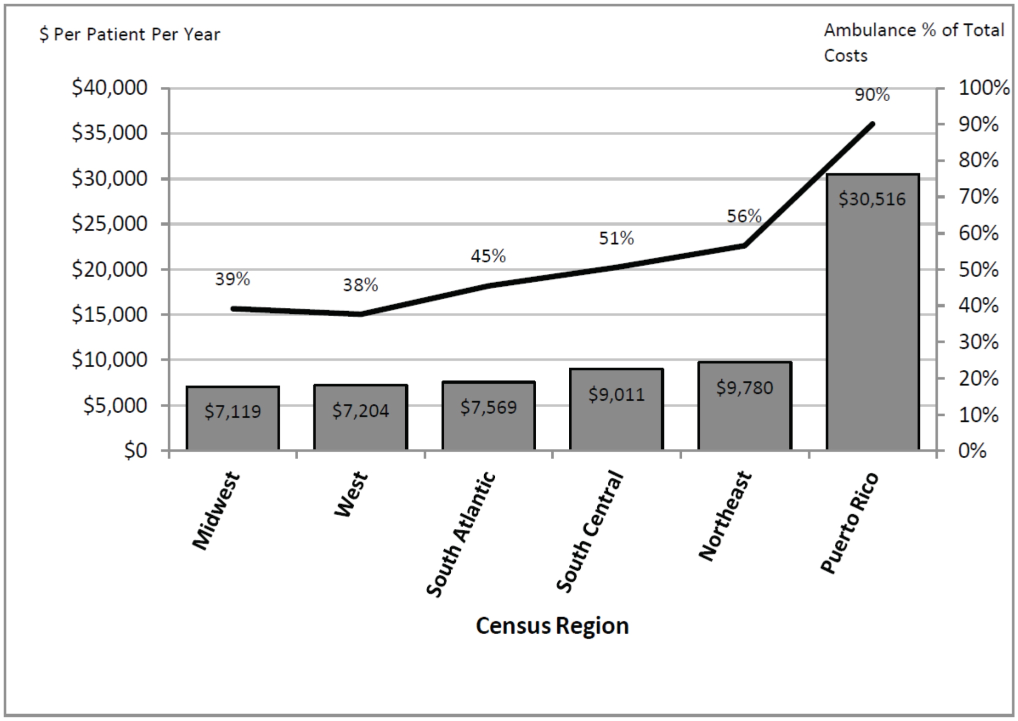

## Sensitivity Analysis

Figure 3 shows the results of the one-way sensitivity analysis. The largest effects on the model cost estimates resulted from the assumptions of utilization mix by type of transport. The range of results varied from 16% below baseline to 20% above baseline when the utilization patterns of individual US regions were substituted for the overall US average pattern. The average cost per trip for NEMT services, the level of insurer discounts from charges, and the average miles driven per trip also showed large effects. Other variables had very small effects by comparison.

**Figure 3.** Sensitivity Analysis: Key Factors Influencing Cumulative Dialysis Transportation Costs from 2012 to 2014

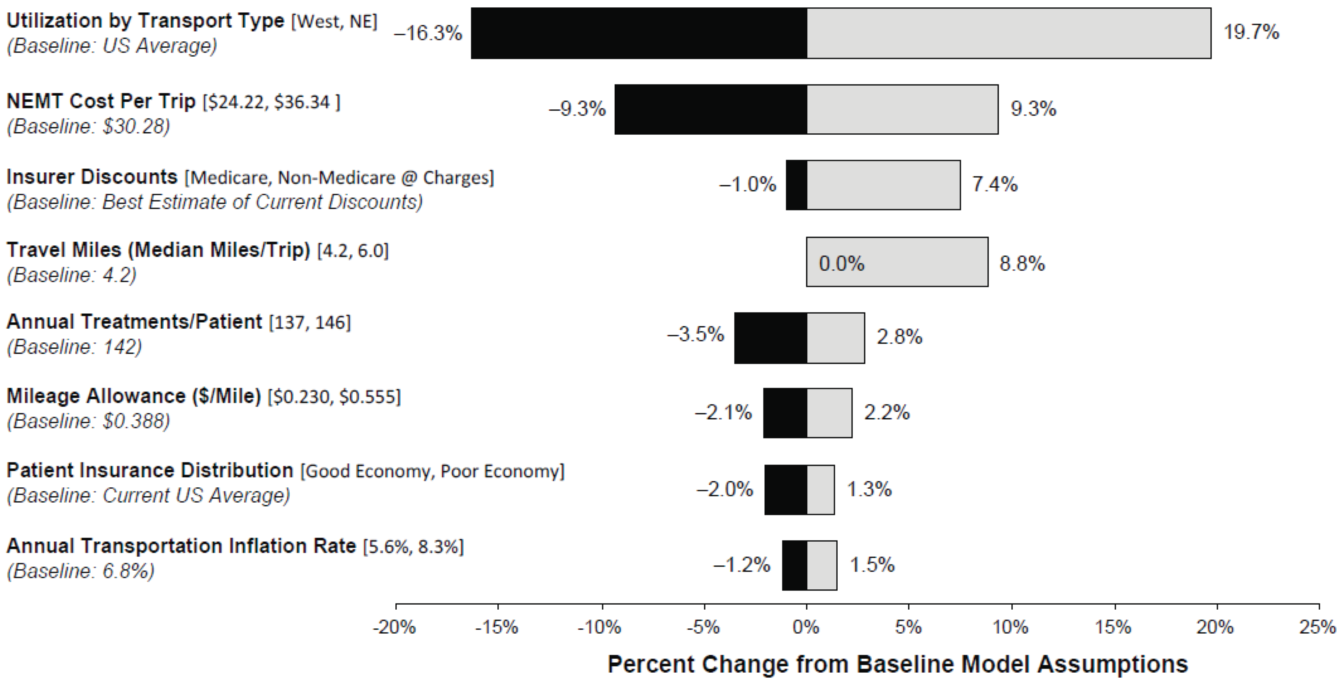

Black bars represent outcomes based on minimum plausible values. Gray bars represent outcomes based on maximum plausible values. Numbers in square brackets to the right of the labels denote the range of values used.

## Savings Estimates

Potential cost savings that may be achieved over 3 years under various scenarios of changes in service utilization mix are shown in Table 8. Changing the mix of services within the four non-ambulance commercial transport categories (ambulettes, wheelchair vans, taxis and public transit) to a lower cost mix of 5% ambulettes, 25% wheelchair vans, 60% taxi and 10% public transit would save nearly $1 billion, or 10% of total costs over 3 years, versus the baseline utilization mix in the model. Shifting patients from costly ambulance transport to less expensive modes of transport offers the potential for dramatic savings. If ambulance use dropped to 1% of trips from the current 5% of trips, the savings would be over $3.2 billion, or one-third of total costs, over the 3-year period. Combining the utilization shifting strategies of the first two scenarios could save $4.2 billion, or 44% of total costs over 3 years.

**Table 8.** Cost Savings Scenarios ($ Billions)

| Scenario | Total Costs 2012-2014 | Savings ($ Billions) | Savings (%) |
|---|---|---|---|
| Baseline | $9.643 | | |
| Reduce intensity of commercial NEMT mix | $8.699 | $0.944 | 10% |
| Reduce ambulance to 1% of trips | $6.418 | $3.225 | 33% |
| Reduce ambulance use and NEMT mix | $5.428 | $4.215 | 44% |

NEMT=non-emergency medical transportation

## DISCUSSION

This study estimated that the costs of transport to routine dialysis for US patients are approximatley $3.0 billion annually and growing rapidly, especially Medicare ambulance costs. The study further showed that if regional or state-level "best practices" were implemented nationally, costs could be cut by one-third or more, saving billions of dollars over 3 years. Despite the apparent high costs of dialysis transportation, this cost segment has received very little attention relative to its overall impact on ESRD costs. For example, concern over the high costs of dialysis drugs arguably led to an entire re-design of Medicare's payment system for dialysis.[22] Yet the costs of ambulances for ESRD patients were rising at a much faster rate than dialysis drug costs and rapidly approached $1 billion per year by 2009 (Table 9).

**Table 9.** Total Medicare Payments ($ Millions) for ESRD Patients: by Select Claim Type[7]

| | 2005 | 2006 | 2007 | 2008 | 2009 | Annual Growth Rate 2005 to 2009 |
|---|---|---|---|---|---|---|
| Total Medicare | $19 687 | $21 657 | $22 641 | $25 131 | $25 115 | 6.9% |
| Outpatient dialysis and injetable drugs | $6284.7 | $6585.7 | $6739.8 | $6957.1 | $7245.8 | 3.8% |
| Ambulance | $580.7 | $701.0 | $758.7 | $842.0 | $929.8 | 15.0% |

In response to the 2012 MedPAC report on Medicare payment for ambulance services,[12] Congress took action in the American Taxpayer Relief Act of 2012 (the so-called "Fiscal Cliff " act) to attempt to restrain the use of ambulances for dialysis transport. The new law cut fee schedule payments for non-emergency basic life support services involving transport of an individual with ESRD to a dialysis facility by 10%, effective October 1, 2013.[23]

Reduced payments should be a disincentive to ambulance providers to transport dialysis patients, but coverage of any alternative means of transport was not addressed. A major policy issue that should be addressed is to determine how to reduce transportation costs without shifting the cost burden to the patient, to the dialysis provider, or to tax-funded public transportation programs. There is already a heavy burden of costs borne by patients, their families and public transit services. This study estimated that approximately one-third of total costs are not covered by patients' primary or secondary insurance. Patients who do not have access to public transportation assistance programs may face a large financial burden due to transport to and from dialysis three times per week. Access to needed services may be reduced or eliminated for some patients, purely based on costs.

Citing budget constraints and the growing demand for dialysis transport, some community transportation services are already turning away dialysis patients.[24] Not only is the prevalent dialysis patient population growing, but also there is growing evidence for the health benefits of short daily hemodialysis versus traditional three-times weekly treatment.[25–28] Added to this is a trend toward more off-hours appointment times, especially at the centers run by the largest dialysis organizations. These factors, taken together, could precipitate more service cutbacks or shortfalls for budget-constrained county and municipal paratransit services. The transportation that many dialysis patients rely on may no longer be available. Concern in some states over this pending crisis of increasing demand in the face of shrinking budgets is high enough that congressmen are taking action to address the problem. At least two bills were introduced in 2012 (H.R. 6011 and S. 2163) to address barriers in accessing quality care for people with kidney failure, including transportation issues.[29]

Policy issues, such as Medicare's limited transportation benefits, and budget cuts to state Medicaid programs and county and municipal transit services, may combine to create a serious crisis in care for dialysis patients. One opportunity to address the issue is through the Dual Eligible demonstration projects funded by the Affordable Care Act. With at least half of the 50 states expected to participate, the ability to coordinate transportation benefits between Medicare and Medicaid for dialysis patients could save a lot of money, and more importantly, protect vital access to medical services for dialysis patients. Another opportunity may result from the ESRD demonstration projects that are under consideration by the Centers for Medicare and Medicaid Services (CMS). In a recent Open Door Forum held by CMS on ESRD demonstration projects, several participants commented on the need to address transportation services.[30]

This study was primarily limited by the lack of quality and the incompleteness of the data used to generate estimates. We are unaware of any systematic reporting on dialysis transportation costs in the United States. There are unreported costs, such as data on the number of patient-driven miles; and information on transportation type used by dialysis patients is very limited. The cost estimates in this study appear to be conservative, when compared with other available literature, and may have underestimated the actual costs by 10% or more. Some model assumptions were generalized across geographic regions, where variation may exist. This limits the reliability of the detailed estimates at the state or region level.

Few studies have been published that attempt to examine overall US dialysis transportation costs and utilization across all transport types and payers. This study begins to explore the range of probable costs and associated policy issues in dialysis transportation. However, higher quality data are needed to more thoroughly address this largely ignored but growing segment of ESRD costs. There are significant savings opportunities that will improve quality of care and protect patient access. Study of these opportunities deserves more attention from policy-makers, especially in the current environment of tightening federal, state, and local budgets that fund much of this care.

**Acknowledgements**

The authors wish to thank Holly Tomlin (employee and stockholder, Amgen Inc.) and Mandy Suggitt on behalf of Amgen Inc. for their medical writing, editing and journal formatting assistance.

**Conflict of Interest Declaration**

Amgen Inc. provided funding for this study, and the manuscript was provided for legal, medical and biostatistical review prior to submission. All content decisions were made at the discretion of the authors. Employees of Amgen Inc. participated in the study design and interpretation of the data, and in reviewing, revising and providing final approval of this manuscript.

JMS has received consulting, travel reimbursement, research, and data collection/analysis fees from Amgen Inc. SB received funds from Prima Health Analytics. SCD has received consulting fees from Amgen Inc and fees from Amgen Inc., NxStage, DaVita, Takeda, Gilead, and Ortho-McNeil. LCE has received consulting fees and travel reimbursement fees from Amgen Inc. DTG has received consultancy fees from Amgen Inc. and research funding from Amgen Inc., NxStage, DaVita, Takeda, and Gilead. ACM has received consulting fees from Amgen Inc. and Abor Research Collaborative for Health.
